# Serum NMR metabolomics uncovers multiple metabolic changes in phenobarbital-treated dogs

**DOI:** 10.1007/s11306-021-01803-5

**Published:** 2021-06-02

**Authors:** Claudia Ottka, Corinna Weber, Elisabeth Müller, Hannes Lohi

**Affiliations:** 1 PetBiomics Ltd., Helsinki, Finland; 2 Department of Veterinary Biosciences, University of Helsinki, Helsinki, Finland; 3 Department of Medical and Clinical Genetics, University of Helsinki, Helsinki, Finland; 4 Folkhälsan Research Center, Helsinki, Finland; 5 LABOKLIN GmbH & Co KG, Bad Kissingen, Germany

**Keywords:** Anticonvulsant, Epilepsy, Metabolism, Phenobarbital, Canine

## Abstract

**Introduction:**

Phenobarbital is a commonly used anticonvulsant for the treatment of canine epileptic seizures. In addition to its central nervous system (CNS) depressing effects, long-term phenobarbital administration affects liver function. However, broader metabolic consequences of phenobarbital treatment are poorly characterized.

**Objectives:**

To identify metabolic changes in the sera of phenobarbital-treated dogs and to investigate the relationship between serum phenobarbital concentration and metabolite levels.

**Methods:**

Leftovers of clinical samples were used: 58 cases with phenobarbital concentrations ranging from 7.8 µg/mL to 50.8 µg/mL, and 25 controls. The study design was cross-sectional. The samples were analyzed by a canine-specific ^1^H NMR metabolomics platform. Differences between the case and control groups were evaluated by logistic regression. The linear relationship between metabolite and phenobarbital concentrations was evaluated using linear regression.

**Results:**

Increasing concentrations of glycoprotein acetyls, LDL particle size, palmitic acid, and saturated fatty acids, and decreasing concentrations of albumin, glutamine, histidine, LDL particle concentration, multiple HDL measures, and polyunsaturated fatty acids increased the odds of the sample belonging to the phenobarbital-treated group, having a *p*-value < .0033, and area under the curve (AUC) > .7. Albumin and glycoprotein acetyls had the best discriminative ability between the groups (AUC: .94). No linear associations between phenobarbital and metabolite concentrations were observed.

**Conclusion:**

The identified metabolites are known to associate with, for example, liver and CNS function, inflammatory processes and drug binding. The lack of a linear association to phenobarbital concentration suggests that other factors than the blood phenobarbital concentration contribute to the magnitude of metabolic changes.

**Supplementary Information:**

The online version contains supplementary material available at 10.1007/s11306-021-01803-5.

## Introduction

Phenobarbital is a barbiturate drug commonly used as an anticonvulsant in the treatment of canine epileptic seizures. The antiepileptic function of phenobarbital is mainly mediated by postsynaptic inhibition via GABA_A_-receptors (Twyman et al., 1989). Phenobarbital prolongs the opening of postsynaptic chloride channels evoked by gamma-aminobutyric acid (GABA), hyperpolarizing the cell membrane, and thus reducing neuronal excitability (Twyman et al., 1989). When used in larger than antiepileptic doses, phenobarbital works as a sedative by inhibiting calcium uptake at nerve endings (Heyer & Macdonald, 1982), and inhibiting the release of the neurotransmitters acetylcholine, norepinephrine, and glutamate (de Boer et al., 1982). The most common form of phenobarbital treatment in dogs is long-term treatment using daily oral dosage, although it is also used intravenously in acute cases.

In addition to its actions as a central nervous system (CNS) depressant, phenobarbital causes multiple metabolic alterations, especially during long-term treatment. Extended phenobarbital treatment causes widespread changes in liver function, affecting the expression of over 50 different genes in liver cells (Frueh et al., 1997). The activity of multiple hepatic enzymes, such as alanine aminotransferase (ALT), alkaline phosphatase (ALP), and gamma-glutamyl transferase (GGT), is often increased during phenobarbital treatment (Müller, Taboada, et al., 2000; Müller, Wolfsheimer, et al., 2000). Increased hepatic metabolism affects for example circulating thyroid hormone levels by decreasing total and free thyroxine, and increasing thyrotropin (Müller et al., 2000; Müller, Wolfsheimer, et al., 2000). Since phenobarbital causes changes in liver function, hepatotoxicity is a possible adverse effect of phenobarbital treatment, especially when using high phenobarbital doses with serum phenobarbital concentrations higher than 40 μg/mL (Dayrell-Hart et al., 1991).

The activation of hepatic enzymes, such as cytochrome P450, also affects the metabolism of multiple drugs, causing phenobarbital treatment to have multiple drug interactions. One of the most important drug interactions is the altered metabolism and increased clearance of phenobarbital, itself (Ravis et al., 1989). This may cause serum phenobarbital levels to fall below the advised therapeutic range during the course of treatment. Thus, serum phenobarbital levels are recommended to be evaluated regularly (Podell et al., 2016).

Although it is known that phenobarbital treatment has widespread metabolic effects, metabolomics studies of the downstream effect of phenobarbital treatment are scarce. This study aims to evaluate the presence of systemic metabolic effects of phenobarbital treatment in dogs using a targeted nuclear magnetic resonance (NMR) metabolomics approach and to discuss the potential significance of these findings on treatment safety and efficacy.

## Materials and methods

**Fig. 1:**
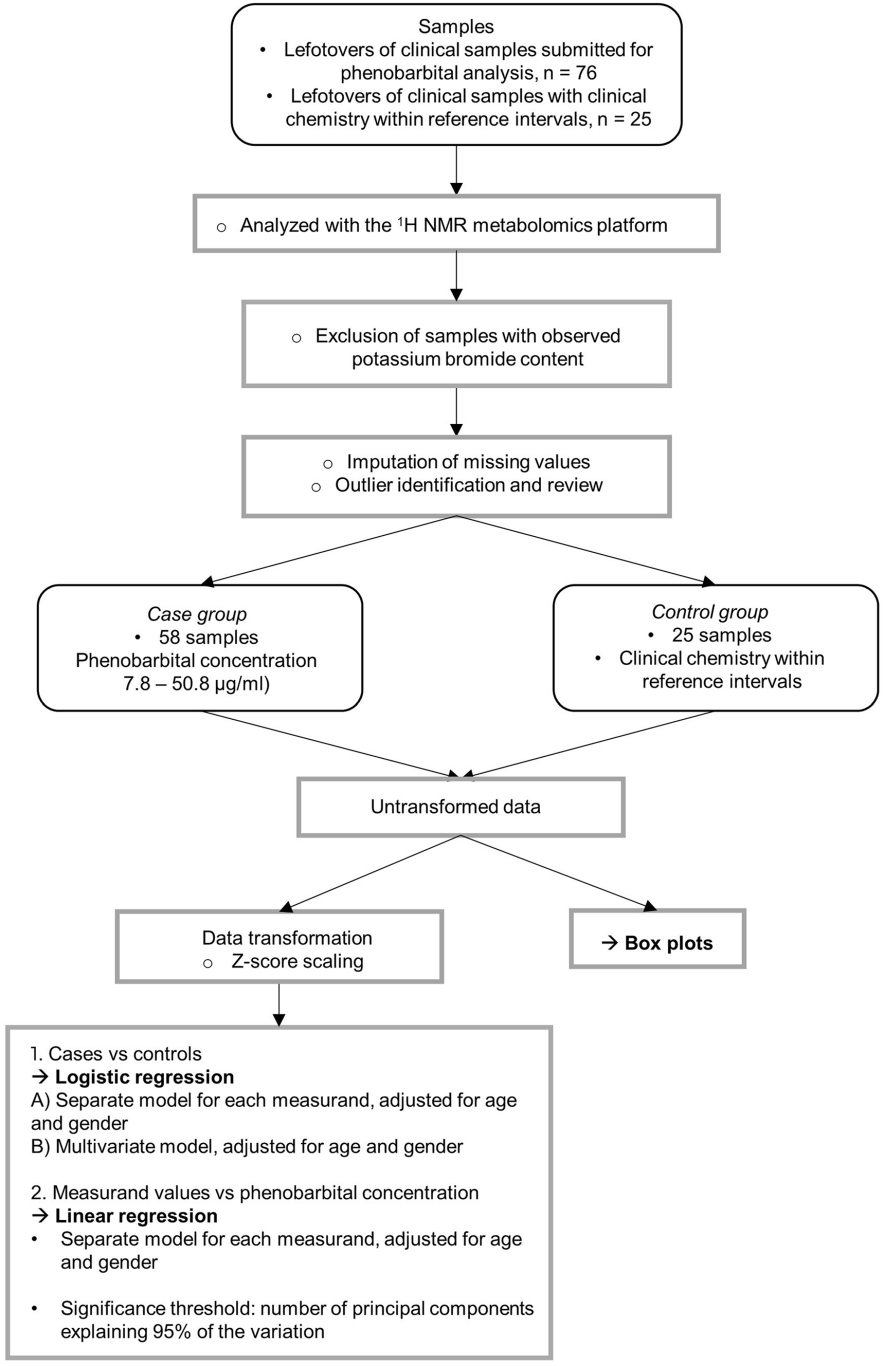
Study workflow. Rounded boxes include information on materials, boxes information on methods. Black points represent sample characteristics, and circles represent data handling procedures. Arrows represent statistical analyses

The workflow of the study is presented in Fig. 1. The study was performed as a case–control study using leftovers of clinical serum samples.

### Samples

The samples consisted of sample material that was left over from laboratory analyses ordered in the patient’s benefit and submitted to a single laboratory provider (Laboklin GmbH & Co KG, Bad Kissingen, Germany). Signalment data limited to age, breed, and sex were available for the samples, but no clinical information or information on sample handling before and during shipment to the laboratory was available.

The case group was based on 76 serum samples submitted for the determination of serum phenobarbital concentration. To reduce the possible confounding effects of potassium bromide medication, we excluded samples, where the determination of potassium bromide concentration was also ordered. The control group consisted of 25 leftover serum samples with standard clinical chemistry within reference intervals.

Upon arrival to the laboratory, leftover sample material was aliquoted and stored at −20 °C for up to 14 days, and shipped to NMR analysis on cool packs. The samples were analyzed by a validated, quantitative, canine-specific ^1^H NMR-based metabolomics platform quantifying 123 measurands with level 1 metabolite identification (Ottka et al., 2019). The fatty acids included in the platform are quantified as serum total fatty acids, including fatty acids in their esterified form. Fatty acid quantification using this method does not require additional sample pretreatment.

### Statistical analysis

The data were evaluated for outliers outside two standard deviations of the mean, and the need for their exclusion was reviewed. Missing values were imputed with the mean and the data were scaled to standard deviation (SD) units using z-score scaling.

**Fig. 2:**
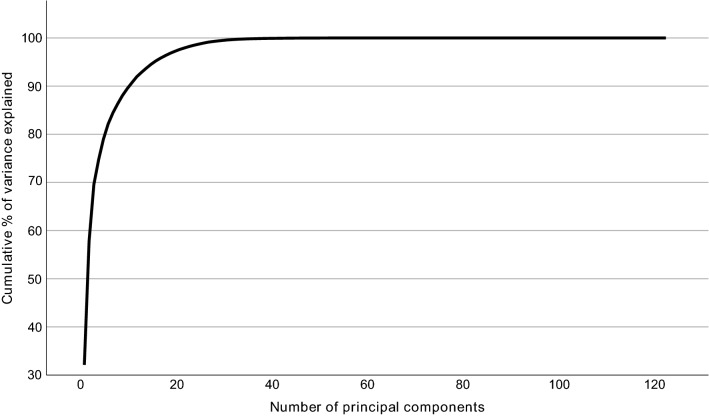
Cumulative percentage of variance explained by each component in principal component analysis

Due to the intercorrelation of the measurands, Bonferroni correction was conducted based on the number of principal components explaining 95% of the variation in the data (Wurtz et al., 2017), a method which has become standard practice in the field. The justification behind this procedure is, that since Bonferroni correction is designed for independent tests, it is too stringent for intercorrelated measurands (Wang et al., 2015). However, principal component analysis can be used to break the intercorrelated measurands into uncorrelated variables (Wang et al., 2015). Fifteen principal components explained 95% of the variation in the data (Fig. 2), resulting in a significance threshold of *P* < 0.0033 (0.05/15).

To determine measurand association with phenobarbital treatment, we created separate logistic regression models for each measurand. The case–control status served as the response variable and the individual metabolite as the dependent variable. All models were adjusted for age and sex. We used the odds ratio and its’ confidence intervals (CI) to determine the strength of measurand association to phenobarbital treatment. Since the odds ratio corresponds to the change in odds of the outcome per one unit of the variable, and since the data was scaled to SD units, a one-unit increase in the odds ratio corresponds to a change in metabolite concentration in the magnitude of one standard deviation. Predictive value of the models with significant (*P* < 0.0033) measurand association to phenobarbital treatment was assessed using the area under the curve (AUC). A high (≤ 1) AUC indicates better predictive value of the model, with 0.7 being the threshold for fair, 0.8 for good, and 0.9 for an excellent model.

In clinical practice, patient results are evaluated in untransformed units against reference intervals. We created box plots using the untransformed data to visualize the differences between the original values in phenobarbital-treated dogs and controls. To visualize whether changes in measurand concentrations in phenobarbital-treated dogs would be visible in clinical practice based on reference intervals, we included serum reference intervals of the NMR method (Ottka et al., 2019) to the box plots.

We performed multivariate logistic regression with forward stepwise selection to determine the measurands with the highest discriminative ability between phenobarbital-treated dogs and controls while adjusting for age and sex. First, we added the measurand with the smallest *p*-value in the logistic regression models created for single measurands. Then, the measurand with the second smallest p-value was added. This procedure was repeated until no additional measurands gained statistical significance (*P* < 0.0033).

We fitted linear regression models for all measurands to assess, whether metabolic changes are linearly associated with the serum phenobarbital concentration. The serum phenobarbital concentration served as the response variable, individual metabolites as dependent variables, and each model was adjusted for age and sex. The significance threshold was *P* < 0.0033.

Statistical analyses were carried out using IBM SPSS Statistics for Windows, version 25 (IBM Corp., Armonk, N.Y., USA) and Microsoft Office Excel, Microsoft Corp., Redmond, WA, US.

## Results

### Sample characteristics

We studied the metabolic changes associated with phenobarbital treatment using a case group of leftovers of clinical samples submitted for serum phenobarbital concentration determination. Seven case group samples had glucose concentrations under 2.5 mmol/L, and three had missing glucose results with lactate over 5.5 mmol/L. These samples were removed from further analyses. The resulting case group consisted of 58 samples with serum phenobarbital concentrations ranging from 7.8 µg/mL to 50.8 µg/mL, with a mean of 21.7 µg/mL (Table 1). The control group consisted of 25 samples with standard clinical chemistry within reference intervals. The age and sex distributions were similar in both groups. Both groups consisted of a large variety of different breeds, with no single breed dominating in either group (Table 2). Mixed breed dogs accounted for 25.9% of the case group, and 24.0% of the control group samples. The remaining case group samples consisted of samples from 21 different breeds, and the control group consisted of samples from 16 different breeds. Breed was unknown for three control group samples and one case group sample.

**Table 1:** Sample characteristics

	*n*	Mean phenobarbital (range) µg/mL	Mean age years (range)	% Males/females
Controls	25	NA	7 (1–15)	64/36
Cases	58	21.7 (7.8–50.8)	7 (1–14)	60/40

Sample characteristics of the case (*n* = 58) and control (*n* = 25) groups. Sex was unknown for 5 case group and 3 control group samples, and age was unknown for 3 case group samples, and 1 control group sample

**Table 2:** Breed distributions in the case and control groups

Cases (*n* = 58)		Controls (*n* = 25)	
Breed	%	Breed	%
Mixed breed	25.9	Mixed breed	24.0
Labrador retriever	8.6	Unknown	12.0
Poodle	6.9	Akita inu	4.0
Yorkshire terrier	6.9	Beagle	4.0
Chihuahua	5.2	Bearded collie	4.0
French bulldog	5.2	Boston terrier	4.0
Australian shepherd	3.4	French bulldog	4.0
Beagle	3.4	German shepherd	4.0
Bolonka zwetna	3.4	Giant dane	4.0
Cairn terrier	3.4	Irish terrier	4.0
Dachshound	3.4	Islandic hound	4.0
German shepherd	3.4	Magyar vizsla	4.0
Golden retriever	3.4	Miniature schnauzer	4.0
Bolonka Zwetna	1.7	Rhodesian ridgeback	4.0
Border Collie	1.7	Swiss Hound—Jura Hound	4.0
Cavalier King Charles spaniel	1.7	Swiss shepherd	4.0
Maremmo sheepdog	1.7	West Highland white terrier	4.0
Miniature Australian shepherd	1.7	Yorkshire terrier	4.0
Parson Jack Russell terrier	1.7		
Pug	1.7		
Schnauzer	1.7		
Swiss Mountain dog	1.7		
Unknown	1.7		

### Albumin, glycoprotein acetyls, saturated fatty acids, amino acids, and lipoproteins are associated with phenobarbital treatment

We created separate logistic regression models for all 123 measurands to assess, whether measurand concentrations differ between the phenobarbital-treated group and control group (Online Resource 1). Albumin, glycoprotein acetyls, relative concentrations of palmitic acid and total saturated (SFA) and polyunsaturated fatty acids (PUFA), the amino acids glutamine and histidine, as well as several HDL and LDL lipoprotein measures were associated with phenobarbital treatment (*P* < 0.0033) and the predictive values of these models was fair to good (AUC 0.73–0.82) (Table 3). Increasing concentrations of glycoprotein acetyls (OR = 8.9, CI 2.7–29.4), LDL particle size (OR = 3.4, CI 1.6–6.9), and relative concentrations of palmitic acid (OR = 4.6, CI 2.0–10.6) and SFA (OR = 3.2, CI 1.6–6.6) increased the odds of the sample belonging to the phenobarbital-treated group. Conversely, increasing concentrations of albumin (OR = 0.19 CI 0.08–0.43), glutamine (OR = 0.34, CI 0.18–0.64), histidine (OR = 0.42, CI 0.24–0.75), LDL particle concentration (OR = 0.41, CI 0.24–0.72), multiple HDL measures including HDL cholesterol (OR = 0.35, CI 0.19–0.66), and the relative concentration of PUFA (OR = 0.29, CI 0.13–0.64) decreased the odds of the sample belonging to the phenobarbital-treated group. Sex and age were not significantly associated with case–control status in any of the models.

**Table 3:** Measurands associated with phenobarbital treatment in logistic regression after adjusting for age and sex

Measurand	B	SE	P	Odds ratio (CI)	SD	AUC (CI)
Albumin	−1.67	.42	<.001	.19 (.08–.43)	3.1 g/l	.83 (.73–.93)
Glycoprotein acetyls	2.19	.61	<.001	8.9 (2.7–29.4)	.427 mmol/L	.82 (.73–.91)
PUFA %	−1.23	.40	.002	.29 (.13–.64)	3.1%	.73 (.62–.83)
SFA %	1.18	.36	.001	3.2 (1.6–6.6)	1.3%	.75 (.65–.85)
Palmitic acid %	1.52	.43	<.001	4.6 (2.0–10.6)	1.4%	.75 (.65–.85)
Glutamine	−1.07	.32	.001	.34 (.18–.64)	.100 mmol/L	.74 (.64–.86)
Histidine	−.86	.29	.003	0.42 (.24–.75)	.16 mmol/L	.75 (.63—.86)
HDL cholesterol	−1.04	.32	.001	.35 (.19–.66)	1.4 mmol/L	.75 (.65–.87)
L-HDL cholesterol	−.99	.32	.002	.37 (.20–.70)	.6 mmol/L	.74 (.62–.85)
L-HDL esterified cholesterol	−1.02	.33	.002	.36 (.19–.68)	.5 mmol/L	.74 (.63–.86)
XL-HDL particles	−.89	.29	.002	.41 (.23–.73)	1.9 µmol/L	.73 (.62–.84)
XL-HDL esterified cholesterol	−.85	.29	.003	.43 (.24–.75)	.6 mmol/L	.72 (.61 -.83)
LDL diameter	1.22	.37	.001	3.4 (1.6–6.9)	.4 nm	.79 (.68–.90)
LDL particles	−.89	.29	.002	.41 (.24–.72)	.5 µmol/L	.75 (.64–.85)

The utilized significance threshold was *P* < .0033. The odds ratio indicates the strength of measurand association to phenobarbital treatment. Since the data were scaled to standard deviation (SD) units, a one-unit increase in the odds ratio corresponds to one SD of the measurand. The area under the curve (AUC) indicates the predictive value of the model; .7 is the threshold for fair, .8 for good, and .9 for an excellent model. *N* cases = 58, *n* controls = 25. Glutamine results were missing for 2 case group samples. *B* coefficient, *SE* standard error, *P P*-value, *CI* confidence interval, *SD* standard deviation, *AUC* area under the curve, *PUFA* polyunsaturated fatty acids, *SFA* Saturated fatty acids, *HDL* high-density lipoprotein. *L* large, *XL* extremely large, *LDL* low-density lipoprotein. Degrees of freedom = 1

**Fig. 3:**
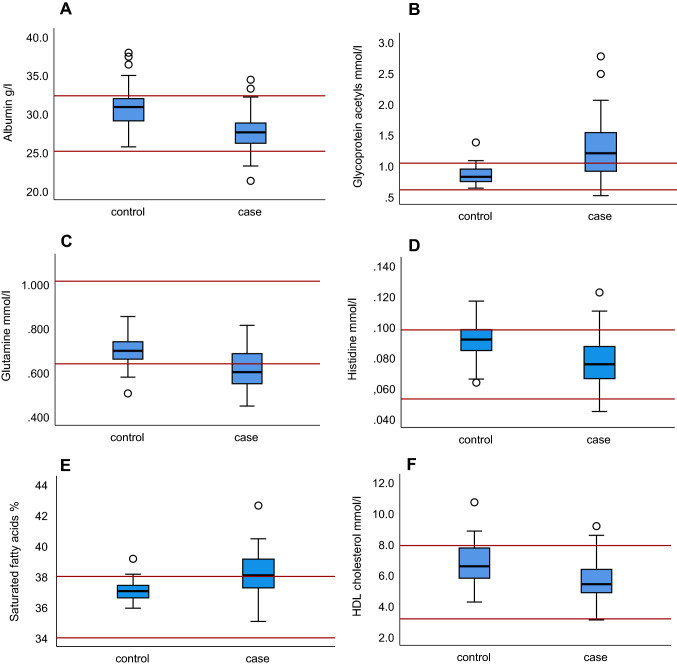
Box plots of untransformed measurand values in the case and control groups for selected measurands associated with phenobarbital treatment in logistic regression. The horizontal red lines represent serum reference intervals of the NMR method (Ottka et al., 2019). *N* cases = 58, *n* controls = 25. *HDL* high-density lipoprotein

Box plots of the untransformed data were created to visualize the observed changes in original values compared to analysis reference intervals (Ottka et al., 2019) (Fig. 3, Online Resource 1). Case group medians of glycoprotein acetyls, total saturated fatty acids %, palmitic acid %, and glutamine were outside analysis reference intervals.

### Albumin and glycoprotein acetyls together have the highest discriminative ability between the phenobarbital-treated group and the control group

**Fig. 4:**
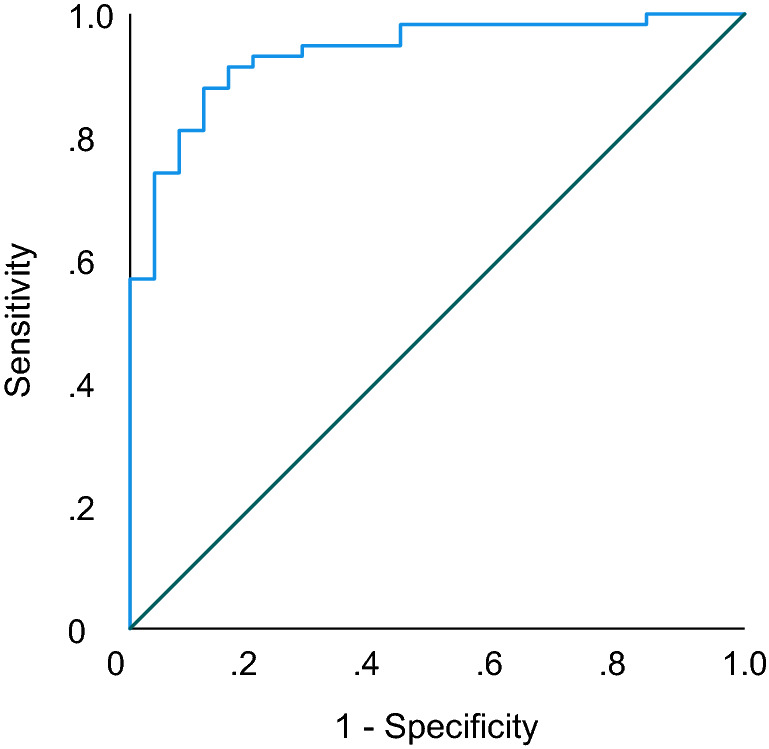
Receiver operating characteristic (ROC) curve of the multivariate logistic regression model including albumin and glycoprotein acetyls, adjusted for age and sex. The area under the curve (AUC) of the model is .94 (CI .88–.99), indicating excellent predictive ability of the model. *N* cases = 58, *n* controls = 25

The model with the best discriminative ability between the phenobarbital-treated group and the control group was determined by performing multivariate logistic regression with forward stepwise selection. Albumin and glycoprotein acetyls were identified as the best discriminators between the case and control groups, while age and sex were insignificant as predictors (Table 4). The receiver operating characteristic (ROC) curve analysis using this model produced an AUC of 0.922, indicatig excellent predictive ability for the model (Fig. 4).

**Table 4:** Results of multivariate logistic regression for the top discriminating metabolites albumin and glycoprotein acetyls, in addition to age and sex

Variable	B	SE	P	Odds ratio (CI)
Age	−.15	.12	.217	.86 (0.68–1.09)
Sex	−.58	.80	.474	.56 (.12–2.72)
Albumin	−2.02	.55	<.001	.13 (.05–.39)
Glycoprotein acetyls	2.79	.82	.001	16.3 (3.2–82.0)
Constant	3.67	1.34	.006	39.2

Since the metabolite data were scaled to standard deviation (SD) units, a one-unit increase in the odds ratio corresponds to one SD of the measurand. *N* cases = 58, *n* controls = 25. *B* coefficient, *SE* standard error, *P P*-value, *CI* confidence interval. Degrees of freedom = 1

### No linear association of measurand results with serum phenobarbital concentration is observed

We created separate linear regression models for all 123 measurands to assess whether a linear relationship exists between serum phenobarbital concentration and the measurand values (Online Resource 1). Significant association (*P* < 0.0033) with serum phenobarbital concentration was not observed for any of the measurands.

## Discussion

Phenobarbital is a commonly used drug in the treatment and prevention of canine epileptic seizures. Phenobarbital treatment is also known to affect metabolism, especially liver function, but its metabolic effects have not yet been extensively studied in metabolomics settings. We utilized a novel, targeted canine-specific NMR metabolomics platform to identify circulating metabolites associated with phenobarbital treatment. We found many physiologically relevant metabolites associated with phenobarbital treatment, including albumin, glycoprotein acetyls, SFA and PUFA as well as the individual saturated fatty acid palmitic acid, amino acids glutamine and histidine, and HDL and LDL lipoprotein measures. The highest predictive ability was observed for albumin and glycoprotein acetyls. These findings provide new insights into the global metabolic effects of phenobarbital treatment, and warrant further studies to identify possible clinical outcomes.

Higher levels of glycoprotein acetyls (GlycA) increased the odds of the sample belonging to the phenobarbital-treated group, and its median concentration was above the reference interval in the case group. GlycA is a novel composite inflammatory marker with the contribution of the signals of the acute-phase proteins α1-acid glycoprotein (AGP), haptoglobin, α1-antitrypsin, α1-antichymotrypsin, and transferrin, and a small contribution of glycosylated apolipoproteins (Otvos et al., 2015). GlycA is considered a marker of systemic inflammation in humans, and has been linked with long-term risk of severe infection, and even mortality (Connelly et al., 2017; Fischer et al., 2014; Fizelova et al., 2017; Otvos et al., 2015; Ritchie et al., 2015). It is known, that the concentration of AGP, a major contributor to the GlycA signal, is elevated in phenobarbital-treated dogs (Hojo et al., 2002), and phenobarbital treatment has been shown to affect the course of the inflammatory process in rats (Levy et al., 1991). Phenobarbital induces AGP gene expression by acting directly on hepatocytes by a pathway independent of inflammation (Fournier et al., 1994). However, this increase has an additive effect with the main inflammatory drivers of AGP gene expression; interleukin-1, interleukin-6, and glucocorticoids (Fournier et al., 1994). In addition to being an acute-phase protein, AGP is a vital carrier molecule, and increased AGP concentrations affect the pharmacokinetics of multiple drugs (Israili & Dayton, 2001; Kremer et al., 1988).

Dogs with lower albumin concentrations were more likely to belong to the phenobarbital-treated group, although overt hypoalbuminemia was rarely reached. Albumin is produced by the liver, and is an important carrier protein, and required for maintaining blood oncotic pressure and acid–base balance (Thrall et al., 2012). The blood albumin concentration decreases during liver failure and chronic inflammatory diseases (Thrall et al., 2012). Long-term phenobarbital treatment reduces serum albumin concentrations (Chauvet et al., 1995). If overt hypoalbuminemia is observed in conjunction with elevated liver enzyme values ALT and ALP, treatment discontinuation is advised due to the possibility of hepatotoxicity (Plumb, 2015).

Circulatory glutamine is largely synthesized in the liver by glutamine synthetase, which catalyzes the glutamine synthesis from glutamate and ammonia (Listrom et al., 1997; van Straaten et al., 2006). Phenobarbital treatment has been associated with the down-regulation of hepatic glutamine synthetase (Klepeisz et al., 2013), which most probably explains why in our study, samples with lower glutamine concentrations were more likely to belong to the phenobarbital-treated group. Glutamine plays an important role in multiple cellular functions in various organ systems, the CNS being one of them (Curi et al., 2005). In neurons, glutamine is utilized for the production of both the excitatory neurotransmitter glutamate and the inhibitory neurotransmitter GABA (Eid et al., 2016). Glutamate-glutamine cycling is considered to contribute to the development of epileptic seizures, with delayed synaptic glutamate clearance causing excitotoxicity (Eid et al., 2016; Flanagan et al., 2018; Petroff et al., 2002). Increased plasma glutamate and decreased plasma glutamine levels have been reported in both juvenile myoclonic epilepsy patients on antiepileptic treatment and in untreated patients with acute tonic–clonic seizures (Rainesalo et al., 2004).

Lower levels of serum histidine increased the odds of the sample belonging to the phenobarbital-treated group. Histidine is an essential amino acid in dogs utilized in multiple important metabolic pathways, including the histaminergic system in the CNS (Bhowmik et al., 2012). Decreased histidine concentrations have previously been observed during acute tonic–clonic seizures and juvenile myoclonic epilepsy in humans (Rainesalo et al., 2004). Intraperitoneal histidine injections have been shown to dose-dependently inhibit myoclonic jerks and tonic–clonic seizures in rats with chemically-induced seizures (Chen et al., 2002). Since histidine injections have been shown to increase histamine concentrations in the cerebral cortex, amygdala and hippocampus of rats (Chen et al., 1999), the seizure inhibiting effect of histidine has been suspected to be caused by increased histamine formation (Chen et al., 2002).

The higher relative concentration of total SFA and the saturated fatty acid palmitic acid, and the lower relative concentration of total PUFA increased the odds of the sample belonging to the case group. However, no significant change in their molar concentrations were observed. This indicates a relative, rather than an absolute change in blood fatty acid composition in phenobarbital-treated dogs. PUFA supplementation has been suggested beneficial for epileptic patients by reducing seizure susceptibility (Al Khayat et al., 2010; DeGiorgio et al., 2015; Reda et al., 2015; Schlanger et al., 2002). However, the determination of plasma fatty acid content has rarely been a part of studies examining the effects of fatty acid supplementation.

Reduced concentrations of multiple HDL measures, and LDL lipids were associated with increased risk of the sample belonging to the case group. However, overt hypocholesterolemia was rarely reached. HDL is the major lipoprotein class for canine cholesterol transport. The liver is responsible for cholesterol biosynthesis (Xenoulis & Steiner, 2010). Hepatic failure can reduce hepatic cholesterol synthesis capacity, and if cholesterol excretion exceeds synthesis, hypocholesterolemia may occur (Thrall et al., 2012). Since hepatic failure is a known, possible adverse effect of phenobarbital treatment (Dayrell-Hart et al., 1991), further studies are needed to evaluate whether lower HDL cholesterol concentrations during phenobarbital treatment are caused by reduced hepatic function.

None of the measurands showed a linear association with the measured phenobarbital serum concentration. This finding suggests, that the magnitude of metabolic changes in phenobarbital-treated dogs cannot be directly derived from the serum phenobarbital concentration. Since phenobarbital induces its own elimination (Ravis et al., 1989), phenobarbital dose and serum concentration might not always be directly proportional. Thus, further research is needed to identify whether the variation in measurand concentrations in phenobarbital-treated dogs would rather be linearly associated with the ingested phenobarbital dose than its serum concentration, or whether they are caused by other physiological or pathophysiological factors.

Several case group samples were removed from statistical analyses due to extreme outlier glucose (<2.5 mmol/L) or lactate (>5.5 mmol/L) concentrations. They were removed, since we suspected a preanalytical cause for these results: prolonged contact to red blood cells (RBC) before serum separation causes glucose catabolism within the sample tube, forming lactate (Ottka et al., 2019). While hypoglycemia itself can cause seizures requiring phenobarbital treatment, phenobarbital concentrations are typically not measured in emergency situations, and thus a physiological cause for these values would be unlikely. Therefore, prolonged contact with RBC was deemed the most likely cause for these values.

The major limitation of this study is the absence of clinical information about the samples. Utilization of the residual sample material of clinical samples is an animal-friendly way of conducting science, with easy access to sample material, enabling the effortless creation of large study groups. However, veterinary laboratories do not gather clinical information, they are best suited for basic research in the metabolomics field, paving the way for further studies where broader clinical data is required. Since no clinical information was available, it is possible, that not all dogs included in the control group are healthy. Although highly unlikely, the control group might include dogs that are even treated with phenobarbital. Uncontrolled preanalytical factors, such as unknown sample handing before sample arrival at the laboratory, and animal-based factors, such as the uncontrolled breed distribution and uncontrolled diet, are additional confounding factors of this study. Thus, further studies with well-defined cohorts are required to verify and expand these findings.

This study is the first metabolomics study to characterize the metabolic changes in phenobarbital-treated dogs. We demonstrated the feasibility of the novel canine serum NMR metabolomics platform to simultaneously quantify and identify multiple drug-induced alterations in the systemic metabolism. The altered metabolic profiles of phenobarbital-treated dogs identify known and new markers involved in multiple important metabolic processes, such as liver and CNS function, inflammatory processes and drug binding, which may contribute to treatment safety and efficacy. A replication study utilizing complete clinical data is needed to confirm and expand the findings in this study. The lack of a linear association of the metabolite levels to serum phenobarbital concentration suggests, that the magnitude of metabolic changes in phenobarbital-treated dogs cannot be directly derived from the serum phenobarbital concentration.

## Supplementary Information

Below is the link to the electronic supplementary material.

Supplementary file1 (XLSX 166 kb)

## Data Availability

The datasets generated during and/or analyzed during the current study are available from the corresponding author on reasonable request.

## References

[CR1] Al Khayat HA, Awadalla MM, Al Wakad A, Marzook ZA (2010). Polyunsaturated fatty acids in children with idiopathic intractable epilepsy: Serum levels and therapeutic response. Journal of Pediatric Neurology.

[CR2] Bhowmik M, Khanam R, Vohora D (2012). Histamine H3 receptor antagonists in relation to epilepsy and neurodegeneration: A systemic consideration of recent progress and perspectives. British Journal of Pharmacology.

[CR3] Chauvet, A. E., Feldman, E. C., & Kass, P. H. (1995). Effects of phenobarbital administration on results of serum biochemical analyses and adrenocortical function tests in epileptic dogs. *Journal of the American Veterinary Medical Association*, *207*(10), 1305–1307. https://www.ncbi.nlm.nih.gov/pubmed/7591923 7591923

[CR4] Chen Z, Li W-D, Zhu L-J, Shen Y-J, Wei E-Q (2002). Effects of histidine, a precursor of histamine, on pentylenetetrazole-induced seizures in rats. Acta Pharmacologica Sinica.

[CR5] Chen Z, Sugimoto Y, Kamei C (1999). Effects of Intracerebroventricular Injection of α-Fluoromethylhistidine on Radial Maze Performance in Rats. Pharmacology Biochemistry and Behavior.

[CR6] Connelly MA, Otvos JD, Shalaurova I, Playford MP, Mehta NN (2017). GlycA, a novel biomarker of systemic inflammation and cardiovascular disease risk. Journal of Translational Medicine.

[CR7] Curi R, Lagranha CJ, Doi SQ, Sellitti DF, Procopio J, Pithon-Curi TC, Corless M, Newsholme P (2005). Molecular mechanisms of glutamine action. Journal of Cellular Physiology.

[CR8] Dayrell-Hart B, Steinberg SA, VanWinkle TJ, Farnbach GC (1991). Hepatotoxicity of phenobarbital in dogs: 18 cases (1985–1989). Journal of the American Veterinary Medical Association.

[CR9] de Boer T, Stoof JC, van Duijn H (1982). The effects of convulsant and anticonvulsant drugs on the release of radiolabeled GABA, glutamate, noradrenaline, serotonin and acetylcholine from rat cortical slices. Brain Research.

[CR10] DeGiorgio CM, Miller PR, Harper R, Gornbein J, Schrader L, Soss J, Meymandi S (2015). Fish oil (n-3 fatty acids) in drug resistant epilepsy: A randomised placebo-controlled crossover study. Journal of Neurology, Neurosurgery, and Psychiatry.

[CR11] Eid T, Gruenbaum SE, Dhaher R, Lee T-SW, Zhou Y, Danbolt NC (2016). The glutamate-glutamine cycle in epilepsy. Advances in Neurobiology.

[CR12] Fischer K, Kettunen J, Wurtz P, Haller T, Havulinna AS, Kangas AJ, Soininen P, Esko T, Tammesoo ML, Mägi R, Smit S (2014). Biomarker profiling by nuclear magnetic resonance spectroscopy for the prediction of all-cause mortality: An observational study of 17,345 persons. PLoS Medicine.

[CR13] Fizelova M, Jauhiainen R, Kangas AJ, Soininen P, Ala-Korpela M, Kuusisto J, Laakso M, Stančáková A (2017). Differential associations of inflammatory markers with insulin sensitivity and secretion: The prospective METSIM study. The Journal of Clinical Endocrinology and Metabolism.

[CR14] Flanagan B, McDaid L, Wade J, Wong-Lin K, Harkin J (2018). A computational study of astrocytic glutamate influence on post-synaptic neuronal excitability. PLoS Computational Biology.

[CR15] Fournier T, Mejdoubi N, Monnet D, Durand G, Porquet D (1994). Phenobarbital induction of alpha 1-acid glycoprotein in primary rat hepatocyte cultures. Hepatology (Baltimore, Md).

[CR16] Frueh FW, Zanger UM, Meyer UA (1997). Extent and character of phenobarbital-mediated changes in gene expression in the liver. Molecular Pharmacology.

[CR17] Heyer EJ, Macdonald RL (1982). Barbiturate reduction of calcium-dependent action potentials: Correlation with anesthetic action. Brain Research.

[CR18] Hojo T, Ohno R, Shimoda M, Kokue E (2002). Enzyme and plasma protein induction by multiple oral administrations of phenobarbital at a therapeutic dosage regimen in dogs. Journal of Veterinary Pharmacology and Therapeutics.

[CR19] Israili ZH, Dayton PG (2001). Human alpha-1-glycoprotein and its interactions with drugs†,‡. Drug Metabolism Reviews.

[CR20] Klepeisz P, Sagmeister S, Haudek-Prinz V, Pichlbauer M, Grasl-Kraupp B, Gerner C (2013). Phenobarbital induces alterations in the proteome of hepatocytes and mesenchymal cells of rat livers. PLoS One.

[CR21] Kremer, J. M., Wilting, J., & Janssen, L. H. (1988). Drug binding to human alpha-1-acid glycoprotein in health and disease. *Pharmacological Reviews*, *40*(1), 1–47. http://pharmrev.aspetjournals.org/content/40/1/1 3064105

[CR22] Levy FE, Chauvelot-Moachon L, Florentin I, Forest M, Poüs C, Fournier C, Giroud JP (1991). Modification of inflammatory processes by phenobarbital in rats. Inflammation.

[CR23] Listrom CD, Morizono H, Rajagopal BS, McCann MT, Tuchman M, Allewell NM (1997). Expression, purification, and characterization of recombinant human glutamine synthetase. The Biochemical Journal.

[CR24] Müller PB, Taboada J, Hosgood G, Partington BP, VanSteenhouse JL, Taylor HW, Wolfsheimer KJ (2000). Effects of long-term phenobarbital treatment on the liver in dogs. Journal of Veterinary Internal Medicine.

[CR25] Müller PB, Wolfsheimer KJ, Taboada J, Hosgood G, Partington BP, Gaschen FP (2000). Effects of long-term phenobarbital treatment on the thyroid and adrenal axis and adrenal function tests in dogs. Journal of Veterinary Internal Medicine.

[CR26] Ottka C, Vapalahti K, Puurunen J, Vahtera L, Lohi H (2019). Characteristics of a novel NMR-based metabolomics platform for dogs. bioRxiv.

[CR27] Otvos JD, Shalaurova I, Wolak-Dinsmore J, Connelly MA, Mackey RH, Stein JH, Tracy RP (2015). GlycA: A composite nuclear magnetic resonance biomarker of systemic inflammation. Clinical Chemistry.

[CR28] Petroff OAC, Errante LD, Rothman DL, Kim JH, Spencer DD (2002). Glutamate-glutamine cycling in the epileptic human hippocampus. Epilepsia.

[CR29] Plumb, D. (2015). *Plumb’s Veterinary Drug Handbook* (8th ed.). PharmaVet Inc.

[CR30] Podell M, Volk HA, Berendt M, Löscher W, Muñana K, Patterson EE, Platt SR (2016). 2015 ACVIM small animal consensus statement on seizure management in dogs. Journal of Veterinary Internal Medicine.

[CR31] Rainesalo S, Keränen T, Palmio J, Peltola J, Oja SS, Saransaari P (2004). Plasma and cerebrospinal fluid amino acids in epileptic patients. Neurochemical Research.

[CR32] Ravis, W. R., Pedersoli, W. M., & Wike, J. S. (1989). Pharmacokinetics of phenobarbital in dogs given multiple doses. *American Journal of Veterinary Research*, *50*(8), 1343–1347. https://www.ncbi.nlm.nih.gov/pubmed/2782717 2782717

[CR33] Reda DMA, Abd-El-Fatah NK, Omar TE-SI, Darwish OAH (2015). Fish oil intake and seizure control in children with medically resistant epilepsy. North American Journal of Medical Sciences.

[CR34] Ritchie SC, Wurtz P, Nath AP, Abraham G, Havulinna AS, Fearnley LG, Sarin A-P, Kangas AJ, Soininen P, Aalto K, Seppälä I, Raitoharju E, Salmi M, Maksimow M, Männistö S, Kähönen M, Juonala M, Ripatti S, Lehtimäki T, Jalkanen S, Perola M, Raitakari O, Salomaa V, Ala-Korpela M, Kettunen J, Inouye M (2015). The biomarker GlycA is associated with chronic inflammation and predicts long-term risk of severe infection. Cell Systems.

[CR35] Schlanger S, Shinitzky M, Yam D (2002). Diet enriched with omega-3 fatty acids alleviates convulsion symptoms in epilepsy patients. Epilepsia.

[CR36] Thrall, M. A., Weiser, G., Allison, R. W., & Campbell, T. W. (2012). *Veterinary Hematology and Clinical Chemistry* (2nd ed.). Wiley-Blackwell.

[CR37] Twyman RE, Rogers CJ, Macdonald RL (1989). Differential regulation of γ-aminobutyric acid receptor channels by diazepam and phenobarbital. Annals of Neurology.

[CR38] van Straaten, H. W. M., He, Y., van Duist, M. M., Labruyère, W. T., Vermeulen, J. L. M., van Dijk, P. J., Ruijter, J. M., Lamers, W. H., & Hakvoort, T. B. M. (2006). Cellular concentrations of glutamine synthetase in murine organs. *Biochemistry and Cell Biology = Biochimie Et Biologie Cellulaire*, *84*(2), 215–231. 10.1139/o05-170 10.1139/o05-170 16609703

[CR39] Wang Q, Kangas AJ, Soininen P, Tiainen M, Tynkkynen T, Puukka K, Ruokonen A, Viikari J, Kahonen M, Lehtimaki T, Salomaa V, Perola M, Davey Smith G, Raitakari OT, Jarvelin M-R, Wurtz P, Kettunen J, Ala-Korpela M (2015). Sex hormone-binding globulin associations with circulating lipids and metabolites and the risk for type 2 diabetes: Observational and causal effect estimates. International Journal of Epidemiology.

[CR40] Wurtz P, Kangas AJ, Soininen P, Lawlor DA, Davey Smith G, Ala-Korpela M (2017). Quantitative serum nuclear magnetic resonance metabolomics in large-scale epidemiology: A primer on -Omic technologies. American Journal of Epidemiology.

[CR41] Xenoulis PG, Steiner JM (2010). Lipid metabolism and hyperlipidemia in dogs. Veterinary Journal (London, England: 1997).

